# Diversity and abundance of leafhoppers in Canadian vineyards

**DOI:** 10.1093/jis/14.1.73

**Published:** 2014-01-01

**Authors:** Julien Saguez, Chrystel Olivier, Andrew Hamilton, Thomas Lowery, Lorne Stobbs, Jacques Lasnier, Brian Galka, Xiangsheng Chen, Yves Mauffette, Charles Vincent

**Affiliations:** 1 Agriculture and Agri-Food Canada, Horticulture Research and Development Centre, 430 Gouin Blvd., Saint-Jean-sur-Richelieu, Quebec, J3B 3E6, Canada; 2 Agriculture and Agri-Food Canada, Saskatoon Research Centre, 107 Science Place, Saskatoon, Saskatchewan, S7N 0X2, Canada; 3 Agriculture and Agri-Food Canada, Eastern Cereal and Oilseed Research Centre, 960 Carling Avenue, Ottawa, Ontario, K1A 0C6, Canada; 4 Agriculture and Agri-Food Canada, Pacific Agri-Food Research Centre, 4200 Highway 97, Summerland, British Columbia, V0H 1Z0, Canada; 5 Agriculture and Agri-Food Canada, Southern Crop Protection and Food Research Centre, 4902 Victoria Avenue North, Vineland, Ontario, L0R 2E0, Canada; 6 Co-Lab R & D, Division of AgCord Inc., 655 Delorme Street, Granby, Quebec, J2J 2H4, Canada; 7 Institute of Entomology, Guizhou University, Guiyang, Guizhou, 550025, P.R. China; 8 Université du Québec à Montréal, 141 President-Kennedy Blvd., Montreal, Quebec, H2X 3Y5, Canada

**Keywords:** Cicadellidae, *Eythroneura* spp., *Macrosteles* spp., *Empoasca* spp., *Vitis* spp.

## Abstract

Leafhoppers (Hemiptera: Cicadellidae) are pests of many temperate crops, including grapevines ( *Vitis* species). Uncontrolled populations can induce direct and indirect damage to crops due to feeding that results in significant yield losses and increased mortality in infected vineyards due to virus, bacteria, or phytoplasmas vectored by leafhoppers. The main objective of this work was to determine the diversity of leafhoppers found in vineyards of the three main Canadian production provinces, i.e., in British Columbia, Ontario, and Quebec. Approximately 18,000 specimens were collected in 80 commercial vineyards from 2006 to 2008. We identified 54 genera and at least 110 different species associated with vineyards, among which 22 were predominant and represented more than 91% of all the leafhoppers. Species richness and diversity were estimated by both Shannon’s and Pielou’s indices. For each province, results indicated a temporal variation in species composition. Color photographs provide a tool to quickly identify 72 leafhoppers commonly associated with vineyards.

## Introduction

In Canada, grapes ( *Vitis* spp.) are grown mostly in Ontario (ON - 7,133 ha), British Columbia (BC - 3,676 ha), and Quebec (QC –612 ha), representing, respectively, 61.2%, 31.6%, and 5.3% of the areas devoted to grapevine production in Canada (11,648 ha) ( Statistics Canada 2012 ). Throughout Canada, grapevines are subject to a large number of climatic (e.g., rainfall, degreedays, microclimates) and non-climatic (e.g., landscape, soil type) factors and agricultural practices (e.g., grapevine pruning or hilling, pesticide treatments, ground covers). Consequently, grapevine cultivars, pests, and diseases differ across major grape growing areas. In a given province, some species can represent a risk of damage or pathogen transmission on grapevine that requires management, while in another province the presence of the same leafhopper species can be sporadic and may not require action.

Vineyards can be attacked by a number of arthropods (e.g., cutworms, thrips, phylloxera, mites, mealybugs, beetles, moths), including leafhoppers (Hemiptera: Cicadellidae) ( Bournier 1976 ; Bostanian et al. 2003 ; Bostanian et al. 2012 ). Leafhoppers are piercing-sucking insects that essentially feed on leaf tissues and fluids. Uncontrolled leafhopper populations can seriously damage leaves, resulting in loss of chlorophyll and premature leaf abscission. If left unchecked, severe yield losses due to shrivelling of fruit may occur ( Martinson et al. 1997 ). Furthermore, honeydew excreted by leafhoppers stains the grapes and alters fruit and wine quality. Leafhoppers are also important vectors of plant pathogens, including viruses, bacteria (e.g., *Xylella fastidiosa* , the agent of Pierce’s disease), and phytoplasmas that induce diseases, such as grapevine yellows ( Weintraub and Beanland 2006 ; Musetti 2008 ; Olivier et al. 2012 ). Two economically important phytoplasma diseases have been detected in Canadian vineyards: Bois Noir ( Rott et al. 2007 ) and Aster Yellow ( Olivier et al. 2009 , 2014).

As mentioned above, agricultural practices can impact the biodiversity and abundance of leafhoppers in vineyards. For instance, several plant species are present around the vineyards, and groundcover can be composed of different grasses and/or weeds. Theses plants may serve as hosts for several leafhopper species and as a reservoir of pathogens, such as phytoplasmas or viruses, that can occasionally be transmitted to grapevine.

There are ca. 21,000 species of leafhoppers worldwide ( Oman et al. 1990 ), and ca. 1,088 species occur in Canada ( Maw et al. 2000 ). Biodiversity studies conducted in two commercial vineyards in QC from 1997 to 1999 found 59 leafhopper species associated with vineyards ( Bostanian et al. 2003 ). Worldwide, there have been few studies on biodiversity, distribution, and abundance of leafhoppers in vineyards ( Bonfils and Schvester 1960 ; Bosco et al. 1997 ; Boukhris-Bouhachem et al. 2007 ; Dér et al. 2009 ). Descriptions of leafhopper species associated with vineyards are frequently part of studies on the arthropod fauna of vineyards (e.g., Bostanian et al. 2003 ; Böll and Herrmann 2004 ; Altieri et al. 2005 ; De Valpine et al. 2010 ). In Europe, several studies have focused on *Scaphoideus titanus* Ball, the vector of Flavescence Dorée ( Boudon-Padieu 2002 ; Lessio and Alma 2004 ; Bressan et al. 2006 ; Dér et al. 2007 ; Orosz and Zsolnai 2010 ). In North America, research efforts have focused on *Erythroneura* species that cause serious yield losses ( McKenzie and Beirne 1972 ; Jubb et al. 1983 ; Paxton 1990 ; Costello and Daane 2003 ; Prischmann et al. 2007 ) and on the vectors of *Xylella fastidiosa* Wells et al., the pathogen causing Pierce’s disease ( Almeida and Purcell 2003 ; Redak et al. 2004 ; Ringerberg et al. 2010). In Israel, species associated with Grapevine Yellows or Western-X diseases have also been studied ( Klein et al. 2001 ). Other information related to leafhopper biodiversity has been published on websites (e.g., Wilson and Turner 2010 ; Fauna Europea 2012 ; Dimitriev 2012). Lists of leafhopper species are frequently provided without mention of their relative abundance or associated crops.

Our study aimed to acquire knowledge on leafhopper diversity and abundance in vineyards of BC, ON, and QC by: 1) determining leafhopper relative abundance and diversity per province, 2) comparing biodiversity indices and rankings of the most common species throughout the years within each province, and 3) presenting three color plates that should allow quick identification of 72 adult leafhopper species commonly associated with Canadian vineyards.

## Materials and Methods

The study was carried out from 2006 to 2008 in BC and ON and from 2007 to 2008 in QC for a minimum of 10 weeks each year during the growing season of grapevine (i.e., May to October) ( Table 1 ). The periods of sampling varied according to the location of vineyards and the agricultural practices.

**Table 1:**
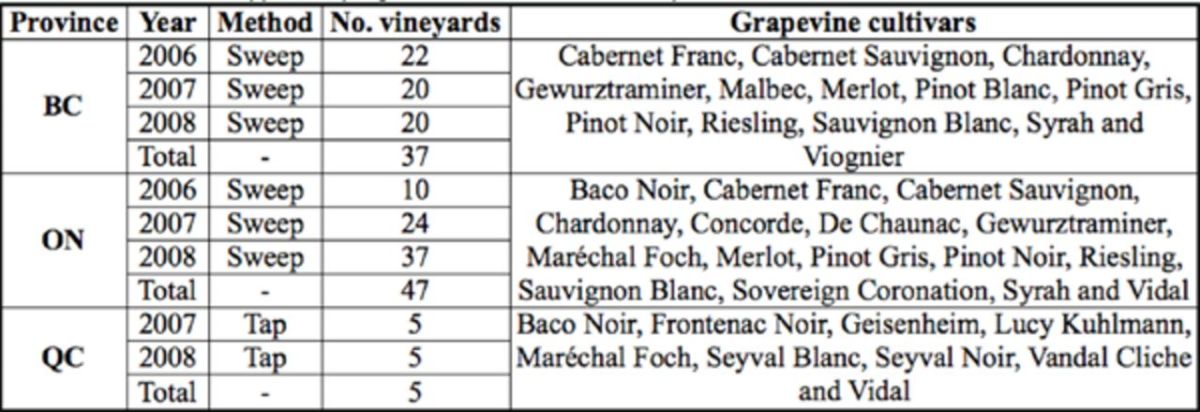
nformation on leafhopper sampling conducted in Canadian vineyards between 2006 and 2008. (74)

### British Columbia

The vineyards were composed of 13 cultivars of *Vitis vinifera* L. (Vitales: Vitaceae) ( Table 1 ), and groundcover was composed of mixtures of grasses and various broadleaf plants between vine rows. Samples were collected each year from 20 to 22 commercial vineyards located throughout the Okanagan Valley, from Vernon in the north to Osoyoos in the south, and in the Similkameen Valley near Cawston and Keremeos ( Table 2 ). Leafhoppers were collected weekly from two rows in each vineyard using the sweeping method (two sets of 25 sweeps, two sweeps/m along the row). Briefly, a 180° cross stroke was followed by a quick backstroke over the same plants to collect flying adult leafhoppers present on groundcovers and on grapevines. Sweep net contents were transferred to clear plastic bags, frozen, and sorted in the laboratory. The specimens were preserved in 70% ethanol or stored dry in 1.5 mL plastic microcentrifuge tubes (Gordon Technologies Inc., Mississauga, ON).

**Table 2.**
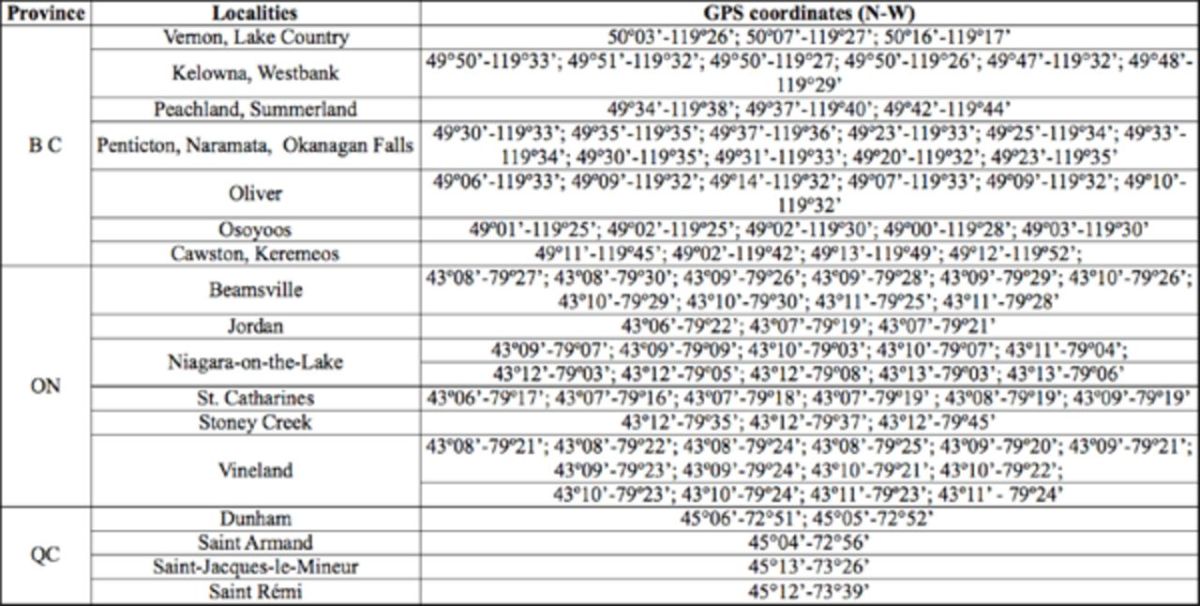
GPS coordinates of vineyard locations in BC, ON, and QC (Canada) between 2006 and 2008.

### Ontario

The vineyards were composed of 16 cultivars ( Table 1 ), and groundcover was composed of various grass species between rows. Forty-seven commercial vineyards located near Beamsville, Jordan, Niagaraon-the-Lake, St. Catharines, Stoney Creek, and Vineland were sampled ( Table 2 ). Leafhoppers were collected weekly from the same five grapevine rows randomly chosen inside the vineyards. The sweeping method (two sweeps/m along the row) was used over a length of 10 m and allowed collection of adult leafhoppers from groundcovers and grapevines. Insects were stored in 70% ethanol and then kept at -20°C until species identification was determined.

### Quebec

The vineyards were composed of nine different cultivars ( Table 1 ), and there was no groundcover between the rows. Surveys were conducted in five commercial vineyards located near Dunham, Saint-Armand, SaintJacques-le-Mineur, and Saint-Rémi ( Table 2 ). Due to the absence of groundcover, adult leafhoppers were collected weekly using a tapping method over 20 m in five grapevine rows randomly chosen in the vineyards. This method consisted of tapping the vines, five times/m at different canopy levels, from the base to the apex of the plant canopy above an aluminum funnel (40 cm diameter) sprayed with 95% ethanol. For each collection in a given vineyard, all leafhoppers were pooled in a tube containing 70% ethanol and stored at - 20ºC.

Leafhoppers were counted and identified in the laboratory using a binocular microscope or sent for identification to the National Insect Collection (Agriculture and Agri-Food Canada). Species were keyed according to several features (e.g., length, morphology, color, genitalia) using Beirne (1956) , Greene (1971) , Hamilton (1982 , 1983, 1998), and Gareau (2008) . Names were cited according to Maw et al. (2000) .

Species richness and diversity between years within a province were estimated by both Shannon’s diversity index and Pielou’s evenness index, as described by Goulet et al. (2004) . The Shannon’s diversity index represents the diversity in a population and was calculated as:

$H^{\prime} = - \sum pi \times \text{ln} \,pi$

where *pi* is the proportion of each species in the global population. The H’ value is a measure of uncertainty that increases with the number of species and when the distribution of specimens among the species becomes more equal. The Pielou’s evenness index represents the evenness of a population. It was calculated as:

$J^{\prime} = H^{\prime} / \text{ln} \,\text{S}$

where S is the number of species collected in the sample. J’ values range between 0 and 1, with high values indicating low variation between species within a given leafhopper population.

## Results

**Figures 1–24.**
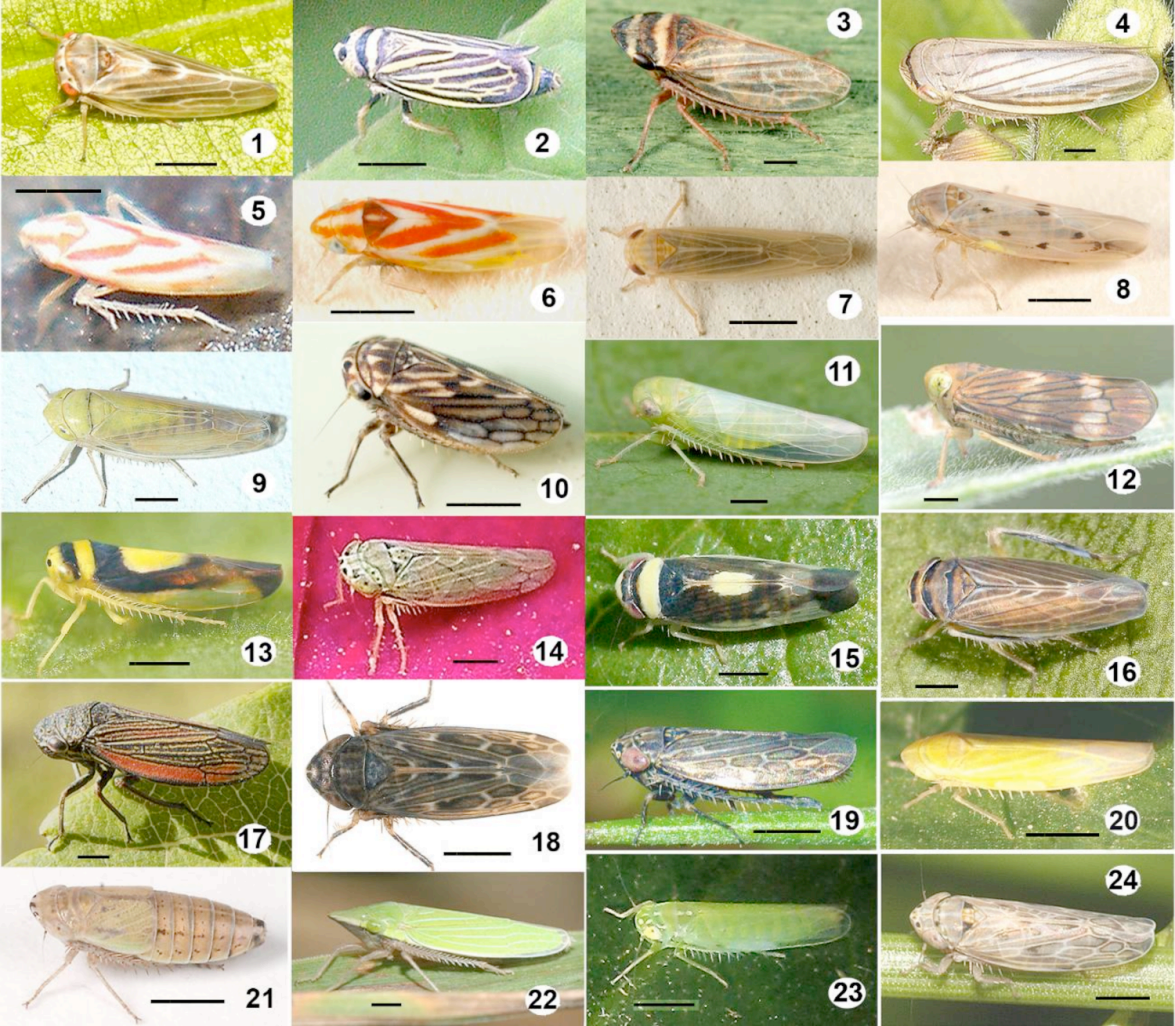
Leafhopper species ( *Agalliopsis* to *Endria)* collected in Canadian vineyards between 2006 and 2008. 1, *Agalliopsis* sp.; 2, *Amblysellus curtisii* ; 3, *Aphrodes* sp.; 4, *Athysanus argentarius* ; 5, *Arboridia dolosa* ; 6, *Arboridia plena* ; 7, *Balclutha neglecta* ; 8, *Balclutha* sp.; 9, *Ballana* sp.; 10, *Ceratagallia* sp.; 11, *Chlorotettix* sp.; 12, *Coelidia olitoria* ; 13, *Colladonus clitellarius* ; 14, *Colladonus geminatus* ; 15, *Colladonus montanus* ; 16, *Colladonus torneelus* ; 17, *Cuerna* sp.; 18, *Deltocephalus grex* ; 19, *Deltocephalus* sp.; 20, *Dikraneura* sp.; 21, *Doratura stylata* ; 22, *Draeculacephala* sp.; 23, *Empoasca* sp.; 24, *Endria inimica.* Approximate scale bar: 1 mm (actual size range listed in Table 3 ). High quality figures are available online.

A total of 17,946 leafhopper specimens were identified in Canadian vineyards between 2006 and 2008 ( Table 3 ). A total of 54 different genera and at least 110 species were identified, belonging to nine subfamilies ( Maw et al. 2000 ). Adults of most species are shown in alphabetical order in Figures 1–72 . About 90% of the collected specimens were identified to species. We assigned the specimens to two categories, i.e., predominant genus (species with > 100 specimens collected over the three years) or marginal genus (species with ≤ 100 specimens). According to our classification, 22 species belonging to 14 genera were predominant and represented > 91% of all the vineyard-associated specimens collected in this Canadian study ( Table 3 ). The most abundant species belonged to the genera *Erythroneura* , *Macrosteles* , and *Empoasca* . The four most common species were *Macro-steles fascifrons* (Stål), *Empoasca fabae* Harris, *Erythroneura comes* (Say), and *E. vitis* (Harris). Because they are well known and major pests in BC vineyards ( Lowery and Judd 2007 ; Lowery 2010 ), only a few *E. ziczac* and *E. elegantula* specimens were preserved for identification.

**Table 3.**
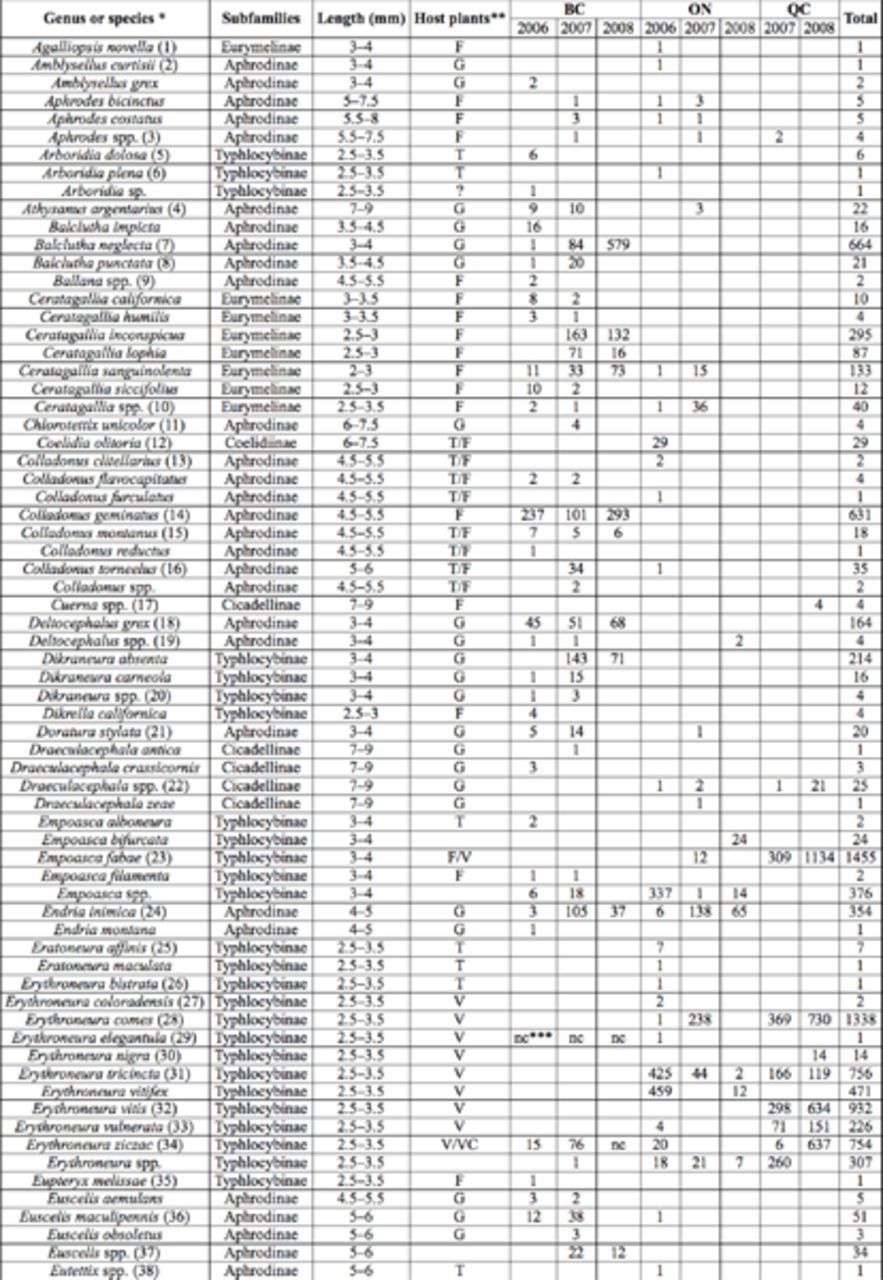

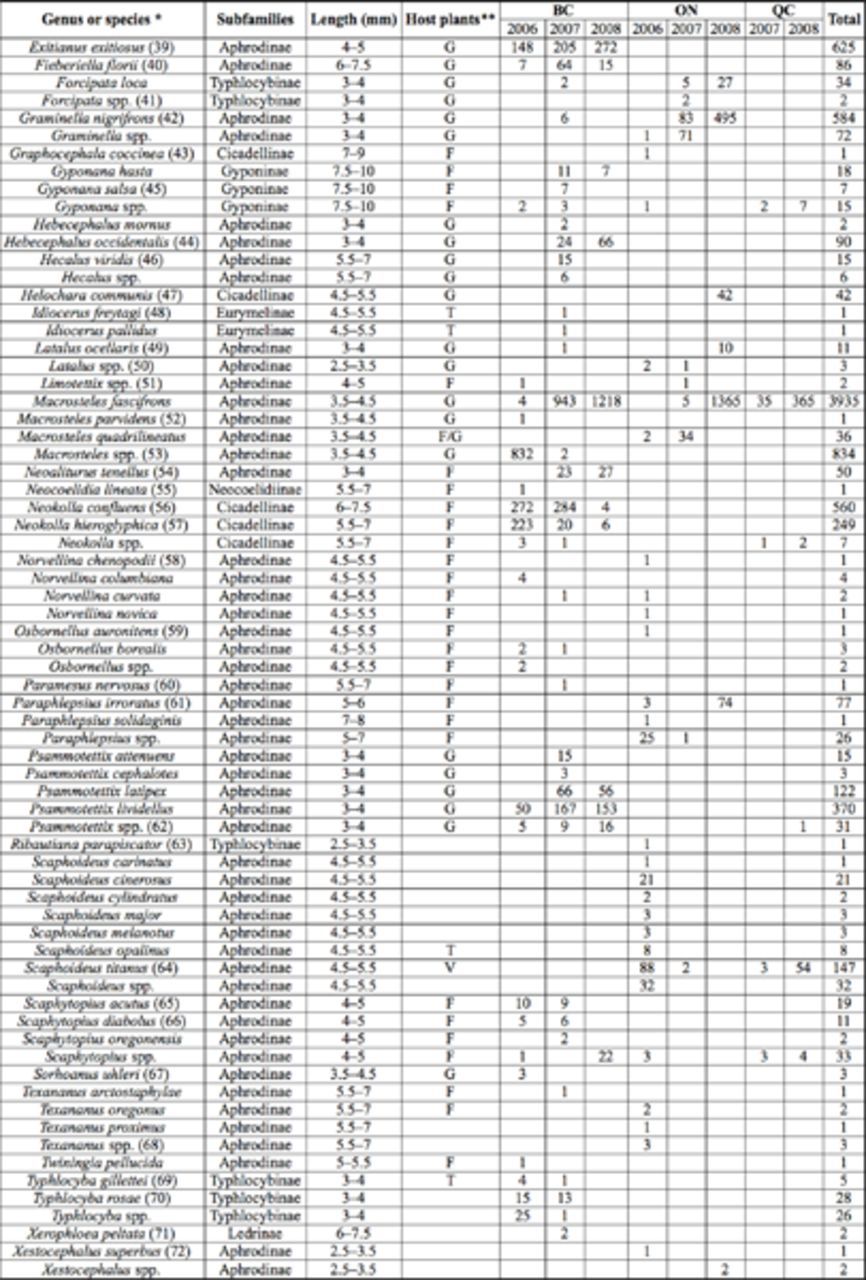
Leafhopper identification and abundance in Canadian vineyards between 2006 and 2008.

*Numbers in parentheses refer to species illustrated in Figures 1–72. ** F: Forbs (herbaceous flowering plants); G: Graminoids (grass, sedges, rushes), T: Trees, V: Vine, VC: Virginia-Creeper. *** nc = not counted

### British Columbia

The 8,146 leafhoppers sampled belonged to 91 different species. Fourteen species were considered predominant, as they represented approximately 88% of all specimens. *Macrosteles* spp. were the most abundant (37%), followed by *Neokolla confluens* (Uhler) and *N. hieroglyphica* (Say) (10%). *Balclutha neglecta* (DeLong and Davidson), *Exitianus exitiosus* (Uhler), *Ceratagallia* spp., *Psammotettix lividellus* (Zetterstedt), and *P. latipex* (Sanders and DeLong) comprised 5–10% of total abundance. Other species (e.g., *Endria inimica* (Say) or *Deltocephalus grex* Oman) represented < 5% of leafhoppers. Several predominant species were collected in all three years of the study.

The total number of species collected increased between 2006 and 2007 and decreased in 2008 ( Table 4 ). The Shannon index (H’) values paralleled the number of species between 2006 and 2008. The Pielou index values (J’) varied from 0.53 to 0.67 between 2006 and 2008 ( Table 4 ), suggesting that species distribution was heterogeneous among years.

**Table 4.**
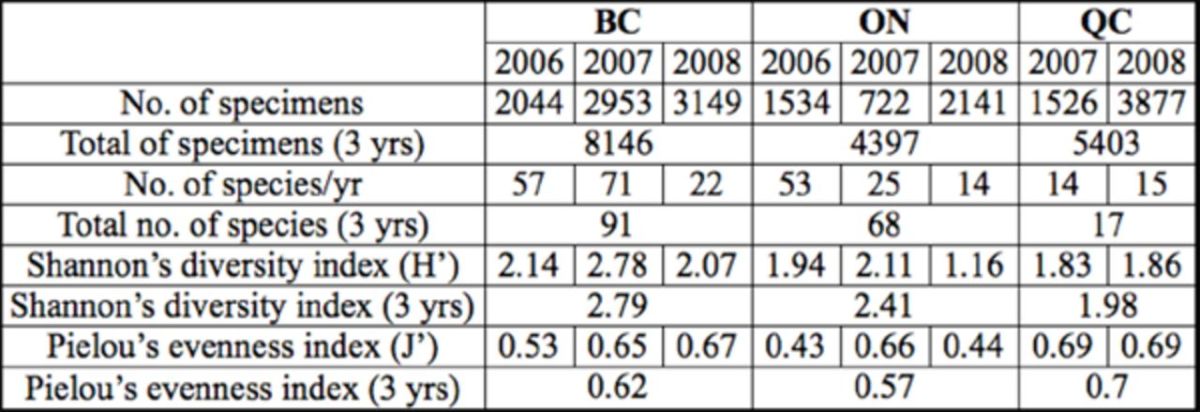
Shannon’s diversity index (H’) and Pielou’s evenness index (J’) of leafhoppers collected in Canadian vineyards from 2006 to 2008.

### Ontario

A total of 4,397 leafhoppers, comprising 68 species, were sampled during the three years. Seven species represented 84% of all leafhoppers. *Macrosteles fascifrons* was most common, representing ca. 31% of all leafhoppers collected in vineyards. Three *EryErythroneura* sp. ( *E. tricincta* Fitch, *E. comes* , and *E. vitifex* Fitch) represented 27% of collected specimens. *Graminella nigrifrons* (Forbes), *Empoasca* spp., and *Endria inimica* were also found in significant numbers. *Scaphoideus titanus* Ball represented 2% of individuals ( Table 3 ).

Diversity and abundance of leafhoppers varied from one year to another. *Empoasca* spp., *E. tricincta* , *E. vitifex,* and *S. titanus* were collected in 2006, *E. comes* and *Endria inimica* in 2007, whereas *Graminella nigrifrons* and *M. fascifrons* were abundant mainly in 2008. *E. vitifex* was only collected in ON. *Endria inimica* and *E. tricincta* were the only species collected each year.

The number of species consistently decreased between 2006 and 2008 ( Table 4 ). However, H’ and J’ values increased in 2007 and decreased in 2008. Because these indices depend on the distribution of the specimens among the species, the variation of H’and J’ values reflected a highly heterogeneous distribution of the specimens within the species between 2006 and 2008 ( Table 4 ).

### Quebec

In 2007 and 2008, 5,403 leafhoppers were collected. They belonged to 17 different species, among which nine were the most common leafhopper species in Canada ( Table 3 ). *Erythroneura* spp. represented > 63% of all leafhoppers collected in 2007 and 2008, with five predominant species, i.e., *E. comes* , *E. vitis* , *E. ziczac* , *E. tricincta* , and *E. vulnerata* Fitch, the latter three species being seldom found in other provinces. The second most abundant genus was *Empoasca* , with *E. fabae* representing 27% of all leafhoppers. *Macrosteles fascifrons* was an abundant species (7%), while *Scaphoideus titanus* represented 1% of specimens. All species mentioned above were collected each year. *Erythroneura vitis* was found only in QC.

Because few species were collected and because these species were similar in 2007 and 2008, the H’ values were low but homogenous ( Table 4 ). Furthermore, the J’ values of evenness were ca. 0.7 in all years, indicating homogeneous distribution over the years.

## Discussion

Leafhoppers had large diversity and heterogeneous abundance in Canadian vineyards. The high heterogeneity in populations between provinces can be explained by different climatic and non-climatic factors and agricultural practices as mentioned above. Consequently, no comparison of diversity and abundance was done between the provinces. Hereafter, we will only focus on the species that have been reported as potential economic pests and may represent a risk to grape production.

*Macrosteles fascifrons* , often confused with *M. quadrilineatus* (Forbes), the six-spotted leafhopper or aster leafhopper, is very common and widespread in Canada ( Beirne 1956 ; Hamilton 1983 ). Our study confirmed its predominance in Canadian vineyards. *Macrosteles fascifrons* is associated with *Juncus bufonis* L. (toad rush) growing in seeps and swales mixed with other low vegetation around vineyards. In contrast, *M. quadrilineatus* disperses by flight over long distances and migrates each year from the USA to Canada ( Taboada and Hoffman 1965 ). *M. quadrilineatus* may feed on grapevine and transmits Aster Yellow (AY) phytoplasmas to numerous plant species ( Beanland 2005 ).

*Erythroneura* species ( Figures 26–34 ) belong to the most abundant leafhopper species in Canadian vineyards and are generally associated with grapevine. *Erythroneura ziczac* ( Figure 34 ) is widely distributed in USA and southern Canada ( Metcalf 1968 ). Our study confirmed that *E. ziczac* was abundant in QC. Because of their abundance and previous description as major pests on grapevines in BC ( McKenzie and Beirne 1972 ; Lowery and Judd 2007 ; Lowery 2010 ), only few specimens of western grape leafhopper ( *Erythroneura elegantula* Osborn, Figure 29 ) and Virginia creeper leafhopper ( *E. ziczac* Walsh) were collected and enumerated in our study.

**Figures 25–48.**
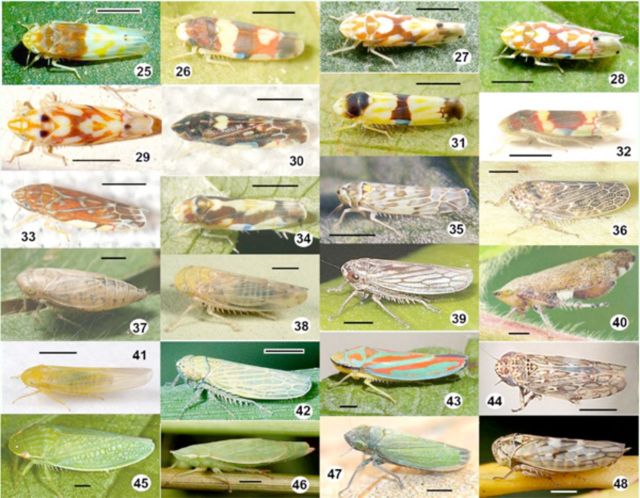
Leafhopper species (Empoasca to Idiocerus) collected in Canadian vineyards between 2006 and 2008. 25, Eratoneura affinis; 26, Erythroneura bistrata; 27, Erythroneura coloradensis; 28, Erythroneura comes; 29, Erythroneura elegantula; 30, Erythroneura nigra; 31, Erythroneura tricincta; 32, Erythroneura vitis; 33, Erythroneura vulnerata; 34, Erythroneura ziczac; 35, Eupteryx melissae; 36, Euscelis maculipennis; 37, Euscelis aemulans; 38, Eutettix sp.; 39, Exitianus exitiosus; 40, Fieberiella florii; 41, Forcipata sp.; 42, Graminella nigrifrons; 43, Graphocephala sp.; 44, Hebecephalus sp.; 45, Gyponana salsa; 46, Hecalus sp.; 47, Helochara communis; 48, Idiocerus freytagi. Approximate scale bar: 1 mm (actual size range listed in Table 3 ). High quality figures are available online.

*Erythroneura comes* (eastern grape leafhopper, Figure 28 ), *E. vitis* (grapevine leafhopper, Figure 32 ), *E. tricincta* (three-banded leafhopper, Figure 31 ), *E. vitifex* , and *E. ziczac* (Virginia creeper leafhopper) may also have economic importance ( Bostanian et al. 2003 ). *Erythroneura* spp. essentially feed on mesophyll, creating stipples on the leaves. Low infestations reduce photosynthesis capacities and consequently reduce sugar production and accumulation in grapes. Heavy infestations can cause complete defoliation of grapevines, inducing plant stunting and serious losses in fruit yield and quality.

*Empoasca fabae* ( Figure 23 ) was one of the most abundant species captured in Canadian vineyards. Migratory adults, airborne on warm spring air currents from the USA, are dispersed over considerable distances ( Hamilton 2010 ). *Empoasca fabae* uses grapevine as a secondary host and causes sporadic injury with little or no economic consequences ( Funt et al. 2002 ). However, heavy infestations can result in serious yield losses, notably in QC where this species was chiefly collected.

*Scaphoideus titanus* ( Figure 64 ) is univoltine and specifically feeds on grapevines. Native to the Nearctic Region, it is widespread in Europe ( Alma 2004 ) and would be likely to settle in Australia, New Zealand, Chile, and South Africa. It may transmit phytoplasma that causes a quarantine grapevine yellow disease of the Elm Yellows group, i.e., Flavescence Dorée. Rapid propagation in plants causes vine death within a few years ( Boudon-Padieu 2000 , 2002). The presence of this leafhopper pest in Canadian vineyards represents an important risk of phytoplasma propagation, even if Flavescence Dorée has not been reported yet in Canadian vineyards ( Olivier et al. 2014 ). *Scaphoideus titanus* was also experimentally demonstrated as a vector of phytoplasmas 16SrI-B causing Aster Yellow disease ( Alma et al. 2001 ). *Scaphoideus titanus* was formerly referred to as *S. littoralis* Ball ( Nielson 1968 ) and is often confused with *S. cyprius* DeLong and Moore, a bog-inhabiting species ( Hamilton 1983 ); females of these species cannot be differentiated.

Species from the genus *Nesosteles* , *Neokolla* ( Figures 56–57 ), *Psammotettix* ( Figure 62 ), *Exitianus* ( Figure 39 ), *Ceratagallia* ( Figure 10 ), *Colladonus* ( Figures 13–16 ), *Deltocephalus* ( Figures 18–19 ), *Dikraneura* , *Endria* ( Figure 24 ), and *Graminella* ( Figure 42 ) are generally found in grasses and forbs; may use grapevines as secondary hosts ( Beirne 1956 ; Nielson 1968 ). Grasses present in groundcovers may constitute reservoir plants for phytoplasmas and may favor the spread of disease to grapevines. *Endria inimica* (painted leafhopper, Figure 24 ) is very destructive to grasslands and can indirectly transmit viruses and Aster Yellow group (16SrI) phytoplasmas ( Wilbur 1954 ; Hill and Sinclair 2000 ). *Neokolla confluens* ( Figure 56 ) and *N. hieroglyphica* ( Figure 57 ) feed on woody plants ( Beirne 1956 ) and weeds, transmit the alfalfa witches’ broom ( Khadhair et al. 1997 ), and are known as vectors of phytoplasmas causing Pierce’s disease and Western X-disease on grapes ( Frazier and Freitag 1946 ). In BC, their nymphs occasionally infest the shoots of grapevines in sufficient number to require control (T. Lowery, personal communication). *Colladonus geminatus* (Van Duzee, Figure 14 ) transmits both AY and Western X-disease to fruit trees. Several species of *Ceratagallia* have similar habits and transmission potential ( Nielson 1968 ). Other species are also known as pathogen vectors on grasses. *Psammotettix alienus* (Dahlbom), a European grass-feeding leafhopper, may be a vector of the persistent wheat dwarf virus. This species causes yellowing and important yield losses (two tons/ha) on wheat ( Lindsten 1980 ). *Graminella nigrifons* ( Figure 42 ) was also described as an important vector of the maize chlorotic dwarf virus ( Nault and Madden 1988 ).

**Figures 49–72.**
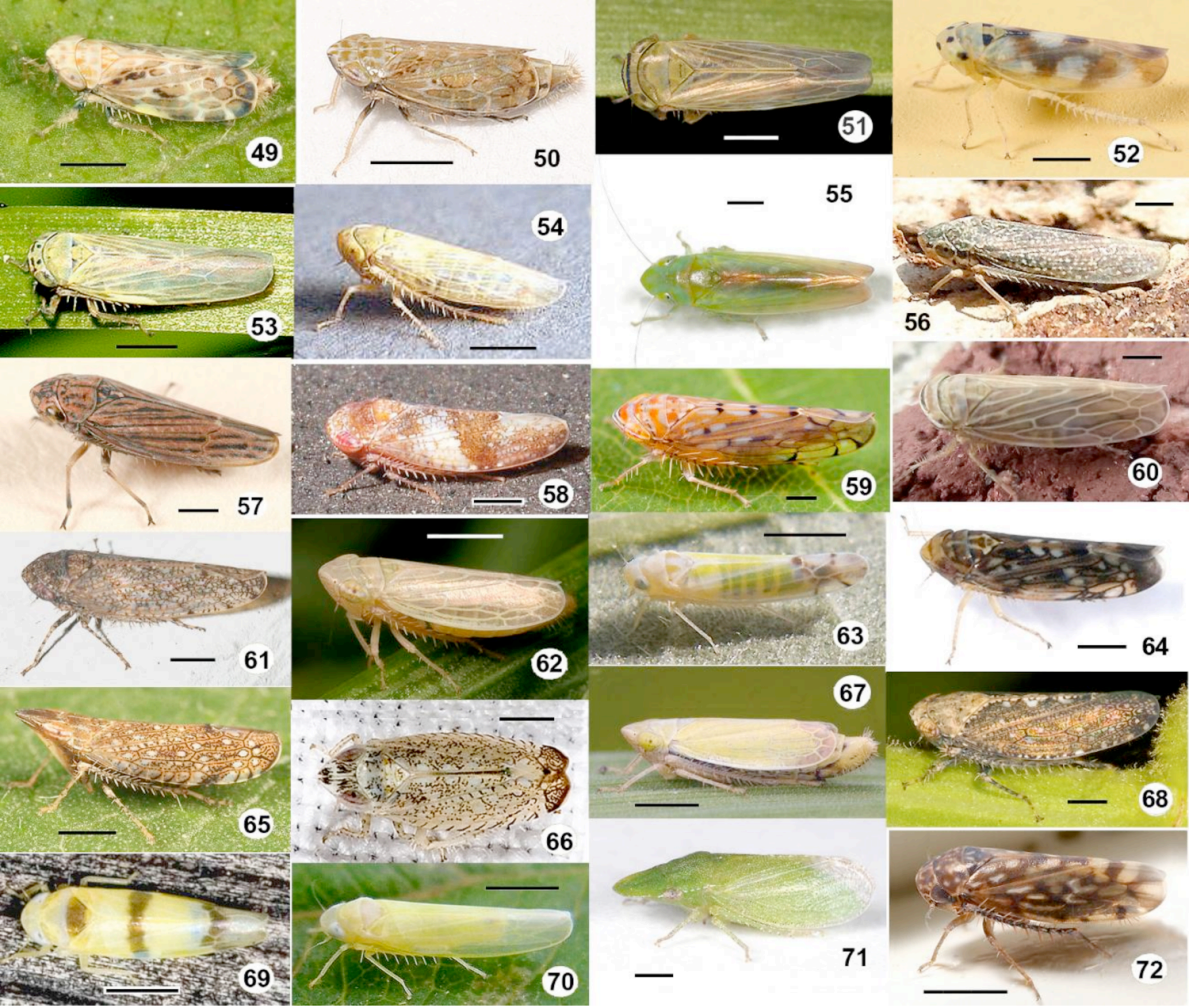
Leafhopper species ( *Latalus* to *Xestocephalus* ) collected in Canadian vineyards between 2006 and 2008. 49, *Latalus ocellaris* ; 50, *Latalus* sp.; 51, *Limotettix* sp.; 52, *Macrosteles parvidens* ; 53, *Macrosteles* sp.; 54, *Neoaliturus tenellus* ; 55, *Neocoelidia* sp.; 56, *Neokolla confluens* ; 57, *Neokolla hieroglyphica* ; 58, *Norvellina chenopodii* ; 59, *Osbornellus auronitens* ; 60, *Paramesus* sp.; 61, *Paraphlepsius irroratus* ; 62, *Psammotettix* sp.; 63, *Ribautiana* sp.; 64, *Scaphoideus titanus* ; 65, *Scaphytopius acutus* ; 66, *Scaphytopius diabolus* ; 67, *Sorhoanus* sp.; 68, *Texananus* sp.; 69, *Typhlocyba gillettei* ; 70, *Typhlocyba rosae* ; 71, *Xerophloea peltata* ; 72, *Xestocephalus superbus.* Approximate scale bar: 1 mm (actual size range listed in Table 3 ). High quality figures are available online.

### Conclusion

We found a large diversity of leafhoppers associated with Canadian vineyards between 2006 and 2008. Several collected species have been described as vectors of pathogens. Because diseases transmitted by leafhoppers can negatively impact grapevines, the development of optimal sampling plans is an important step to their management. The collection of photographs ( Figures 1–72 ) should provide a useful tool for researchers, grape growers, agronomist, technicians, and students to quickly identify leafhoppers in vineyards. Our results suggest that the challenges to manage leafhoppers associated with Canadian vineyards should differ across provinces. To develop appropriate monitoring and treatment programs against the key leafhopper species, two issues should be considered: 1) the relative abundance of leafhopper species in vineyards, and 2) the vectorship (i.e., the capacity to acquire and successfully transmit phytoplasma to grapevine) likely differ across leafhopper species and remains poorly understood.
